# Polymer System Based on Polyethylene Glycol and TFE Telomers for Producing Films with Switchable Wettability

**DOI:** 10.3390/ijms25094904

**Published:** 2024-04-30

**Authors:** Evgeniy Belov, Konstantine Nadaraia, Igor Imshinetskiy, Dmitry Mashtalyar, Lidia Ignatieva, Yurii Marchenko, Ivan Osmushko, Maria Gerasimenko, Sergey Sinebruykhov, Sergey Gnedenkov

**Affiliations:** 1 Institute of Chemistry Far Eastern Branch Russian Academy of Sciences, 690022 Vladivostok, Russia; belov_eal@mail.ru (E.B.); nadaraiakv@mail.ru (K.N.); igorimshin@gmail.com (I.I.); ignatieva@ich.dvo.ru (L.I.); gor_dvo@mail.ru (Y.M.); gifsundsvall@mail.ru (I.O.); gerasimenko.ms00@mail.ru (M.G.); sls@ich.dvo.ru (S.S.); svg21@hotmail.com (S.G.); 2 Department of Nuclear Technologies, Far Eastern Federal University, 690922 Vladivostok, Russia

**Keywords:** switch polymers, supramolecular system, polyethylene glycol, tetrafluoroethylene telomers, IR-spectroscopy, X-ray photoelectron spectroscopy

## Abstract

Today a lot of attention is paid to the formation of thermosensitive systems for biomedical and industrial applications. The development of new methods for synthesis of such systems is a dynamically developing direction in chemistry and materials science. In this regard, this paper presents results of the studies of a new synthesized supramolecular polymer system based on polyethylene glycol and tetrafluoroethylene telomers. The films formed from the polymer substance have the property of switching wettability depending on temperature after heating activation. It has been established that the wettability changes at 60 °C. The contact angle of activated hydrophobic polymer film reaches 143°. Additionally, the system exhibits its properties regardless of the pH of the environment. Based on data obtained by the methods of infrared and x-ray photoelectron spectroscopy, differential thermal analysis and thermal analysis in conjunction with wettability and morphology, a model of the behavior of molecules in a polymer system was built that ensures switching of the hydrophilic/hydrophobic surface state. The resulting polymer system, as well as films based on it, can be used in targeted drug delivery, implantation surgery, as sensors, etc.

## 1. Introduction

The use in modern chemistry of such types of materials as smart polymers opens a wide range of possibilities [1,2,3,4,5,6,7,8,9]. These are new and easy-to-use detectors [1,2,3,4], which change the shape and appearance of the product under the influence of external factors [5], as well as targeted drug delivery agents [6,7,8,9], which can deliver a drug directly to the required area of the body and provide a dosed effect in this area.

Of particular interest also is the ability of several polymers and supramolecular systems based on them to change their properties under the influence of various environmental factors. This can open completely new possibilities. not only for the use of such polymers, but also for the creation of fundamentally new devices. One of the most popular and well-known polymers with properties that change depending on various environmental factors, based on switchable wettability, are materials based on poly(N-isopropylacrylamide) [10,11,12]. This material has long attracted the attention of researchers due to its ability to change its wettability depending on the temperature [10,11] and pH of the environment [11,12]. Such properties of the material have attracted particular interest for the production of drugs for targeted delivery. In addition to the above polymer, there are many polymer materials capable of switching wettability depending on the temperature and pH [11]. Nevertheless, this polymer remains the most widely used substance with similar properties. However, the relative high cost of poly(N-isopropylacrylamide) limits its use. To solve these practical problems, other materials have been used, such as conjugates of polyethylene glycol (PEG) and hydrophobic agents [13,14,15]. It is reasonable to use fluorine-containing polymers with low wettability as hydrophobic components of such systems. Various oligomeric compounds, for example, tetrafluoroethylene telomers, look especially promising in this case. Due to the presence of hydrophilic and hydrophobic parts, the resulting substances form micelles that have switchable properties depending on environmental conditions. At the same time, the substances used in the synthesis process have relatively low cost, and the resulting compounds can be used to produce novel materials. Currently, researchers are faced with the task of creating new, including supramolecular, polymer materials that are sensitive to changes in environmental parameters, and especially temperature.

As mentioned above, one of the most accessible polymers is polyethylene glycol (PEG), which has several important properties that facilitate its use in the field of biomedicine [13,14,15,16]. PEG is water-soluble, has low immunogenicity, high biocompatibility, is easily excreted from the body naturally and, due to its groups, can form conjugates. Moreover, this material also has a low softening point, which strongly depends on the length of the polymer chain. Thus, the idea of using this polymer with hydrophobic substances, for example, tetrafluoroethylene telomers, to obtain supramolecular systems with the ability to change wettability looks promising. However, to the best of our knowledge, such systems were previously obtained only in our previous work [17], which described only the synthesis method and wettability of polymer films. This work presents more detailed studies of the PEG-telomer tetrafluoroethylene (TFE) polymer system; the change in bonds in the resulting system is studied depending on the initial state, after activation and after temperature exposure. In this work, the mechanism for changes in wettability was proposed and described in detail for the first time and the presence of the supramolecular nature of the synthesized compound was proven. In the future, these polymer systems and films based on them may find application as targeted delivery elements, antibacterial layers for implants, sensors, etc.

## 2. Results

### 2.1. Thermal Analysis

Analysis of previous studies shows that polyethylene glycol derivatives change their structure depending on the temperature and pH of the environment [13,16,18]. These properties of the polymer can be used to obtain a material with variable wettability.

**Figure 1:**
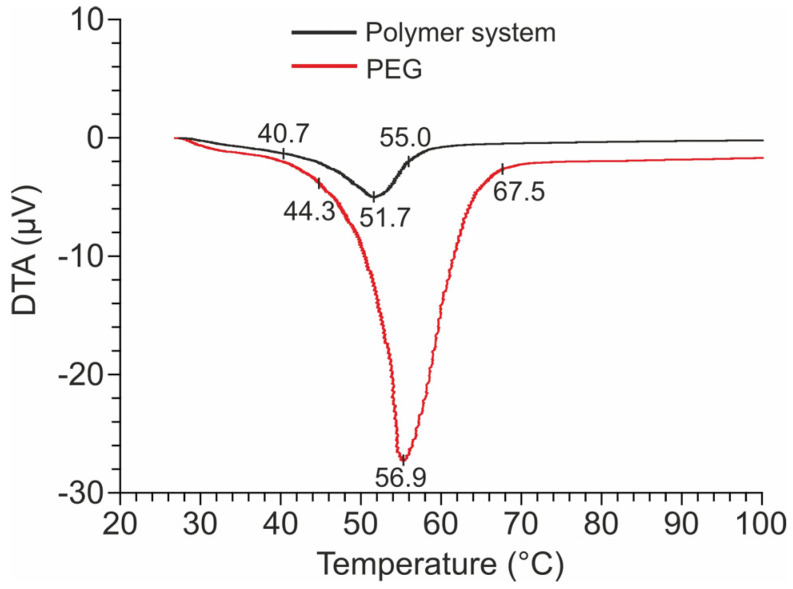
DTA curves of the polymer system and the original polyethylene glycol. DTA data was obtained for the non-activated system.

The temperature behavior of the polymer system was studied using differential thermal analysis (DTA). Figure 1 shows DTA curves for the test substance. During the studies, it was found that the first signs of softening, namely, ongoing changes in the polymer system, are observed at a temperature of 40.7 °C. Additionally, the DTA curve shows an endothermic effect with a minimum at 51.7 °C (Figure 1), due to the softening of PEG, which is the main hydrophilic component of the system. The end of this process is observed at 56.9 °C.

The conditions for activation of the polymer system were determined experimentally during the study of the temperature transition of the film from hydrophilic to hydrophobic state and back. A detailed analysis revealed that changes in film wettability occur at lower temperatures than the primary activation temperature [18]. According to thermal analysis, it was revealed that the activation temperature is near 50 °C. Based on the data obtained, the heating temperature was increased to 70 °C, and the time at this temperature was increased to 5 h. The choice of a temperature of 70 °C for the primary activation of the system was made on the basis of the experiments performed and thermal analysis data. According to the data obtained, the main reactions stopped after reaching 55 °C. However, an attempt to activate the system at a temperature of 60 °C demonstrated the need for too long an exposure time, exceeding 12 h. Therefore, after a number of experiments, we settled on 70 °C for 5 h. At the same time, after activation, the polymer system already demonstrated properties of changing wettability at 60 °C.

From the analysis of the DTA curve of the polymer system, it follows that these temperatures correspond to the beginning and end of the endo effect with a peak at around 50 °C (Figure 1). No other thermal effects were detected up to 150 °C.

### 2.2. Change in Wettability of a Polymer System

Initially, the film of the studied material is hydrophilic. The contact angle of deionized water is close to that of polyethylene glycol but is higher due to the influence of TFE telomers.

**Figure 2:**
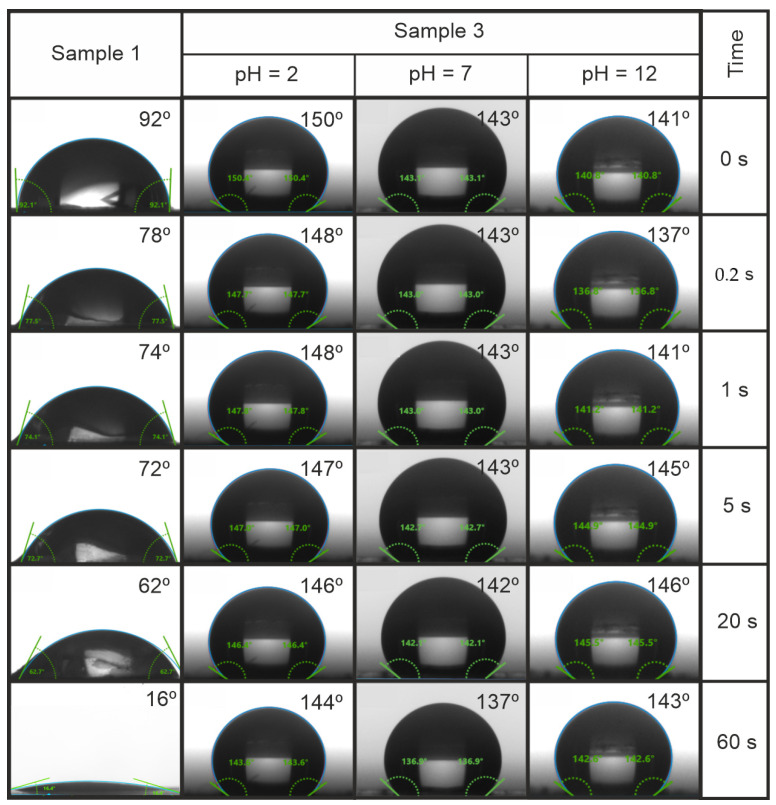
Dynamics of changes in the CA of the film of a non-activated (Sample 1) (at 25 °C) and heated activated (Sample 3) (at 60 °C) polymer system.

When heated to 60 °C, a change in wettability occurs, and the hydrophilic film becomes hydrophobic. The contact angle reaches values of 150° (Figure 2). At the same time, the wettability properties are little affected by the pH. As can be seen from Figure 2, the surface remains highly hydrophobic at 60 °C over time.

An assessment of the surface free energy (SFE) of the resulting polymer system was carried out on samples before thermal activation (Sample 1), after activation (Sample 2) and after switching of properties (Sample 3). It was found that when Sample 1 contacts with water, the main contribution to the SFE is made by the dispersive component (nonpolar interactions). The value of surface energy in this case is 44 mJ/m^2^ (Table 1).

After activation, an increase in the surface energy for the cooled film (Sample 2) to a value of 53 mJ/m^2^ is observed (Table 1). A change in the ratio of the polar and dispersive components of the SFE was revealed. An increase in the polar component is observed compared to the material before activation (Table 1). This increase in the polar component in comparison with sample 1 may be due to the greater mobility of hydrophilic PEG molecules in the system.

For Sample 3 heated to 60 °C, a sharp drop in surface energy to 13 mJ/m^2^ and a decrease in the contribution of the polar component were observed (Table 1). This, in turn, indicates a change in the structure of the polymer under the influence of temperature. The value of the SFE of the film of the activated polymer system is close to this parameter for polytetrafluoroethylene, for which the polar component is also close to zero [19].

If we compare the SFE of films of substances from which the supramolecular system was obtained (PEG and TFE telomers), we can see their mutual influence in the system (Table 1). Thus, the surface energy for the resulting polymer system before activation and after heating to 60 °C (Samples 1 and 2) is closer to the value of this parameter for polyethylene glycol, but noticeably lower (Table 1), which may be caused by the influence of TFE telomers, having low surface energy. The calculated value of SFE for a film formed from a solution of TFE telomers is 6.4 mJ/m^2^, while for the resulting polymer system after activation it is 13 mJ/m^2^. From the data obtained, especially in comparison of the surface energy values for the telomeric film and PTFE [19], we can conclude that the obtained values are due to the features of the surface topography. A similar effect of relief on the value of free surface energy is observed in Sample 3.

### 2.3. Surface Morphology

One of the important features affecting the wettability of a surface is its morphology. Thus, the presence of a hierarchical structure allows the surface to demonstrate superhydrophobic properties.

According to studies of the surface wettability of films obtained based on a polymer system heated after activation, the contact angle reaches 143°, which exceeds the values for the smooth surface of hydrophobic materials. This effect is explained by the influence of the morphology of the resulting film.

**Figure 3:**
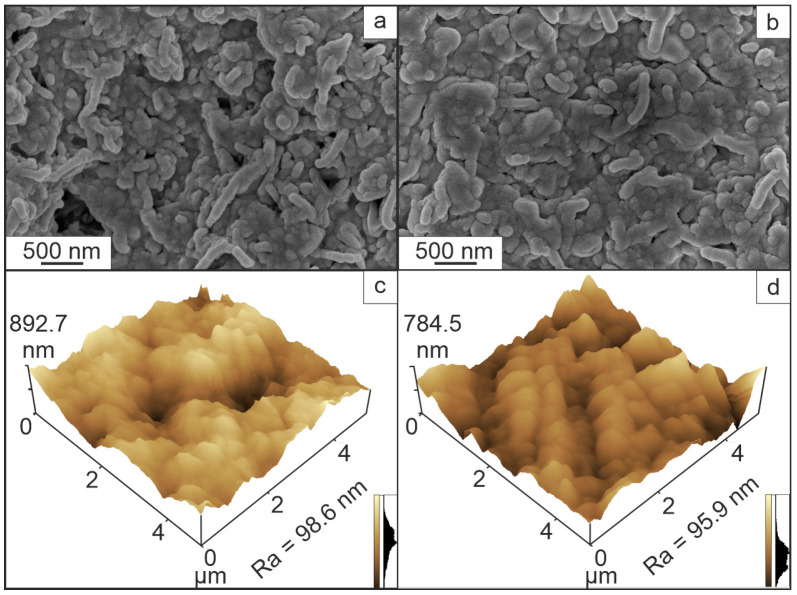
Morphology of the polymeric film of the supramolecular system before (Sample 1, **a**,**c**) and after thermal activation (Sample 2, **b**,**d**). Data obtained by SEM (**a**,**b**) and AFM (**c**,**d**).

The surface microrelief of the film was studied using scanning electron and atomic force microscopy (Figure 3). After solvent removal, the polymer system has numerous structures that form a surface with a developed morphology (Sample 1, Figure 3a,c). After thermal activation of the film, the structural features were preserved, but the microrelief became less developed (Sample 2, Figure 3b,d).

However, assessment of the roughness of the polymeric film before and after activation revealed that its values change slightly after activation of the system.

### 2.4. IR Spectroscopy

IR spectroscopy data obtained for samples of the base substances and synthesized system before and after activation made it possible to identify the features of their molecular structure.

**Figure 4:**
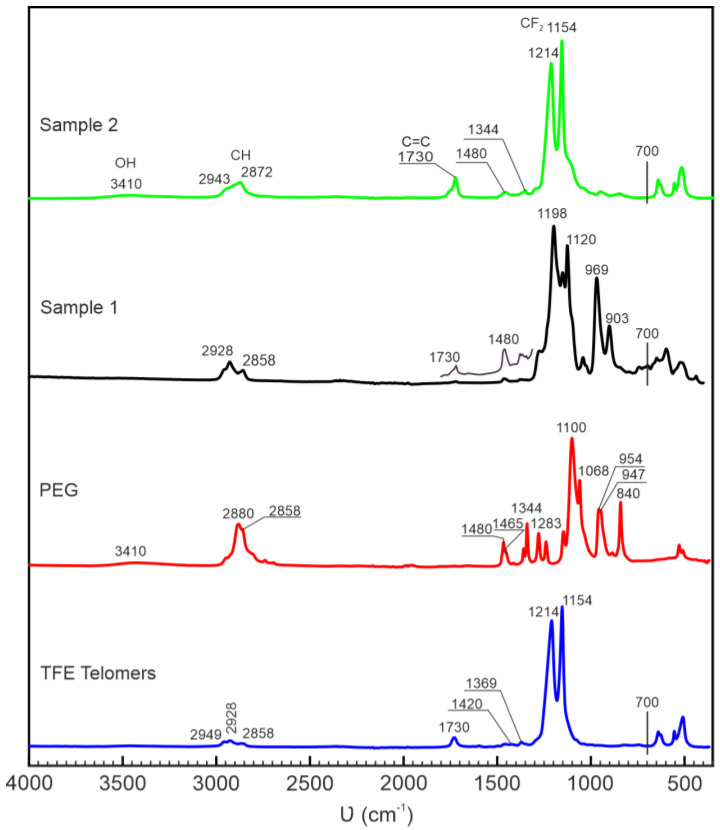
IR spectra of TFE telomers in acetone (blue), PEG (red), and synthesized polymer system before (Sample 1, black) and after (Sample 2, green) activation.

The IR spectrum of TFE telomers obtained in acetone (Figure 4, blue line) is characterized by a number of bands, the most intense of which are the bands at 1214 and 1154 cm^−1^. These bands are always observed in the IR spectra of PTFE and, according to previous studies [20,21], belong to the stretching vibrations of CF_2_ groups. All bands characteristic of PTFE in the low-frequency region of the spectrum (640, 625, 555 and 514 cm^−1^) are clearly visible, which suggests that the molecular structure of TFE telomers is close to the structure of PTFE. Moreover, analysis of bands in the region below 700 cm^−1^ allows us to conclude that telomers have helical structures [22]. In addition to these bands, the spectrum of telomers contains low-intensity bands at 1730, 1420 and 1369 cm^−1^, which, as revealed in [20,21], correspond to vibrations of the terminal groups of formation CH_3_C=OCH_2_–: 1730 cm^−1^ for *ν*(C=O), 1420 cm^−1^ for CH_2_, and 1369 cm^−1^ for CH_3_. The presence of bands at 2858, 2928, and 2949 cm^−1^ is due to the presence of C–H bonds [20]. Based on the intensity ratio *I*_1154_/*I*_1730_, it was established that the molecules of the telomers under discussion contain 14 –CF_2_– fragments. This is consistent with the analysis of the TFE telomer’s length obtained in acetone, presented in [21].

Typically, several characteristic bands are identified in the IR spectrum of PEG, which are used to identify the presence of this polymer [22,23]. Among them is an absorption peak at 1100 cm^−1^, which is responsible for vibrations of ether bonds. The peak at 1068 cm^−1^, which is characteristic of the terminal groups of polyester, is clearly visible. The stretching vibrations of OH groups are represented by a low-intensity broad band with a maximum at 3420 cm^−1^. Stretching vibrations –CH_2_– are characterized by a several bands at 2947, 2880, 2862, 2809 cm^−1^. Deformation and out-of-plane vibrations of –CH_2_– groups are represented by bands at 1344 and 1283 cm^−1^. Absorption bands are detected at 1465 and 1480 cm^−1^, due to the presence of the gauche and trans configuration of the –O–CH_2_–CH_2_–O– group in the polymer. The group of bands below 960 cm^−1^ is characterized by out-of-plane vibrations of –CH_2_– groups.

The IR spectrum of Sample 1 (Figure 4, black) does not represent a superposition of the spectra of individual components (TFE telomers and PEG). On the other hand, the band characterizing the vibrations of the CF_2_ groups of TFE telomers (1154 cm^−1^) is well identified in the IR spectrum of Sample 1. Apparently, the band at 1214 cm^−1^ is also retained, but it is masked by an intense band at 1197 cm^−1^, which is not observed either in the IR spectra of TFE telomers or PEG. The presence of a band at 1154 cm^−1^ in the spectrum suggests the preservation of a fragment of the –CF_2_–CF_2_– chain, characteristic of the molecular structure of the TFE telomers chain. At the same time, it is possible to distinguish low-intensity bands characterizing vibrations of the terminal groups of TFE telomers only with a detailed study. A decrease in their intensity may be associated with chain elongation, for example, during the polymerization of TFE telomers, and, accordingly, a decrease in the number of terminal groups. Other possible explanation is a change in the composition of the terminal groups of TFE telomers obtained in acetone (CH_3_COCH_2_–), due to the addition or incorporation into the structure of another polymer, such as polyethylene glycol. or products formed when substances are mixed. Interestingly, in the IR spectrum of the resulting compound, there are practically no bands, the presence of which can be associated with the presence of the original polyethylene glycol. There is practically no band characterizing the vibrations of OH groups; the band at 1068 cm^−1^, which is attributed to the *ν*(C–OH) vibrations, is not observed either. The vibration region of –CH_2_– groups in the IR spectrum of sample 1 is represented by a group of low-intensity bands in the region of 2950–2850 cm^−1^.

However, the intensity and shape of the band differs markedly from that observed in the IR spectrum of the original PEG. The band at 2880 cm^−1^, usually observed in the IR spectrum of PEG, is not observed in the IR spectrum of Sample 1, which indicates changes in the arrangement of CH_2_ groups in the structure. This is also indicated by the disappearance of the bands at 1465 and 1480 cm^−1^, which are related to the gauche and trans conformations of the –O–CH_2_–CH_2_–O– group.

The IR spectrum of the activated system (Sample 2) is dramatically different from the spectrum of the non-activated system (Sample 1). With the exception of a number of low-intensity bands in the region of 3400 and 3000–2800 cm^−1^, the IR spectrum of the activated system is close to the IR spectra of TFE telomers. The absorption bands characterizing vibrations of –CF_2_– groups (1154 and 1214 cm^−1^) have the highest intensity. It is interesting that all bands in the region below 700 cm^−1^ observed in the IR spectrum of TFE telomers are retained in the IR spectrum of the activated sample. Moreover, bands characterizing vibrations of the terminal groups of telomers are also clearly visible. In the IR spectrum, these are low-intensity bands at 1730, 1459, 1420, and 1369 cm^−1^. In the IR spectrum of the activated sample in this region, these bands are observed: 1722 with a shoulder of 1747 cm^−1^, and 1458 and 1351 with a shoulder of 1366 cm^−1^. The observed splitting of the band in this region of the spectrum (1747 shoulder, 1722 cm^−1^) suggests the presence of a C=O bond in the terminal group in an environment different from that which occurred in the terminal group of the original telomers. Note that, according to the *I*_1154_/*I*_1730_ ratio, the telomers chain length has become shorter (8 CF_2_ fragments). A wide band in the region of C–H stretching vibrations (3000–2800 cm^−1^) in the IR spectrum of the activated sample is represented by a band at 2880 cm^−1^ and overlapping band at 2947 cm^−1^. Both bands were noted when analyzing the IR spectra of PEG, but were not observed in the IR spectrum of Sample 1. In the IR spectrum of the activated sample, like the original PEG, a wide band is visible in the region of 3410 cm^−1^. This is the region in which OH stretching vibrations are located, although the maximum of the band is slightly shifted to the low-frequency region relative to the original PEG. On the low-frequency wing of the band at 1154 cm^−1^, an inflection is visible near 1120 cm^−1^, not associated with vibrations of the fluorocarbon chain. This bending is most likely due to the presence of a band, which, as shown above, can be attributed to one of the *ν*(COC) vibrations of the original PEG.

### 2.5. XPS Analysis

**Figure 5:**
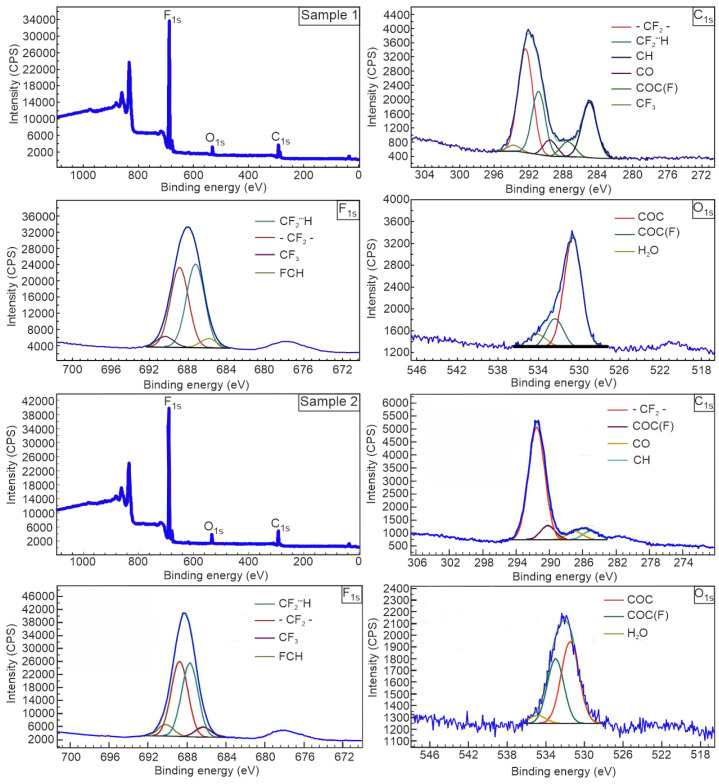
XPS survey and high-resolution spectra for synthesized polymer system before (Sample 1) and after (Sample 2) activation.

The composition of the polymer system and the chemical state of the elements were studied using X-ray photoelectron spectroscopy (XPS). The binding energies and relative group concentrations are presented in Table 2. The survey spectrum (Figure 5) indicates the presence of fluorine (about 689 eV), oxygen (532 eV) and carbon (291 eV). Peaks in the range of 800–900 eV are attributed to Auger satellites represented by secondary electrons of fluorine atoms. The elemental composition is represented mainly by fluorine and carbon.

The F_1s_ line (Figure 5) has a half-width of about 3 eV and a relatively symmetrical contour, which indicates two states of atoms that are close in chemical environment in equal relative abundances. We proposed a decomposition into two main components (688.9 eV and 687.4 eV) for two reasons. The main reason is the influence of the hydrogen bond on the position of the core level of the electronegative atom. As noted in [24], this effect manifests itself in a shift towards increasing the binding energy of the electrons of an electronegative atom oriented towards hydrogen. Thus, the higher binding energy of 688.9 eV can be interpreted as the 1s level component of the fluorine atoms facing the polyethylene glycol chain, i.e., for fluorine atoms participating in the CF∙∙∙H hydrogen bond between TFE telomers and PEG and lying deeper from the surface. Both effects have the same direction of line shift. The binding energy of 687.4 eV indicates the presence of fluorine atoms not bonded by hydrogen bonds in the composition of –CF2– groups.

Note that the quantitative ratio of fluorinated carbon atoms to fluorine atoms is close to the stoichiometry of polytetrafluoroethylene. The state of carbon atoms bound to fluorine is characterized by a binding energy of 292.3 eV and 290.9 eV. This also confirms the presence of CF_2_– groups, partially bonded to hydrogen. Based on the presence of other components of the C_1s_ line, we can speak about the presence of carbon atoms associated with oxygen. The binding energy value of 287.3 eV is due to the presence of a single C–O bond. Possible strongly oxidized states of atoms in the terminal groups R_1_ and R_2_ (C=O, COO), edge and structural effects (FCH)—289.6 eV are allowed. Due to the large number of components and their low intensity, an accurate assessment of relative concentrations is difficult. However, the same states appear in the structure of the F_1s_ and O_1s_ lines. Possible strongly oxidized states of atoms in the terminal groups R_1_ and R_2_ (C=O, COO), edge and structural effects (FCH) are allowed: 289.6 eV. Due to the large number of components and their low intensity, an accurate assessment of relative concentrations is difficult. However, the same states appear in the structure of the F_1s_ and O_1s_ lines.

The noted quantitative and structural features of the material according to XPS data suggest that the surface mainly contains elements of TFE telomer chains located on the surface of PEG globules. In this case, the chains on the surface do not form a continuous layer of telomeric units, leaving areas of the polyester uncovered.

Analysis of high-resolution XPS spectra (Figure 5) of a sample subjected to heat treatment makes it possible to discover new features of the resulting polymer system. Thus, a narrower line of carbon binding energy was identified, assigned to fluorinated carbon (291.5 eV). This indicates one or more energetically similar chemical states of carbon atoms. For this reason, it is difficult to quantify the proportion of carbon found in these states. However, we can point out the presence of a bond between carbon and fluorine with an energy characteristic of an unactivated system (components 290.9 eV and 292.4 eV). In addition, a slight increase in fluorine and a decrease in oxygen and carbon in the CO state in the surface layers were detected. This suggests that, after activation of the polymer system, the fluorine and carbon fractions of the telomers make a greater contribution to the elemental composition.

For Sample 2, a smaller number of impurities (aliphatic carbon, oxygen) was detected. At the same time, a significant decrease in the number of oxygen atoms associated with water is also observed, which, due to the presence of vibrations of OH groups, confirms the presence of terminal groups in PEG.

## 3. Discussion

Based on IR spectroscopy data, it is possible to draw a conclusion about the interaction of the TFE telomers and PEG. At the same time, if in the structure of TFE telomers the fluorocarbon chain (–CF2–)_n_ is generally preserved, then in the structure of polyethylene glycol the changes are more significant. Thus, the bands in the region of 3410 cm^−1^, characterizing the vibrations of –OH groups, are not visible in the IR spectrum of the formed supramolecular system (Figure 4). This gives grounds to assume that there is no noticeable number of OH groups in the structure of the synthesized sample in IR spectroscopy. This is also indicated by the absence of a band at 1068 cm^−1^ (Figure 4), which is attributed to vibrations of the terminal groups *ν*(C–OH). Considering the change in the position of the band at 1100 cm^−1^ (Figure 4), noticeable changes occur in the C–O–C bond. It should be noted that bands at 969 and 903 cm^−1^ appear in the IR spectrum of the polymer system (Figure 4).

When the system is activated, changes occur that lead to the release of TFE telomers chains to the surface. This is precisely why the ATR spectra of the activated sample show high similarity with the spectrum of TFE telomers, and the presence of PEG is reflected only in the presence of low-intensity bands in the region of 2900 cm^−1^ (Figure 4). Note that the structure of the TFE telomers chains, except for the length, is practically unchanged. It can be assumed that the interactions between TFE telomers and PEG are most likely supramolecular.

One of the signs of the presence of supramolecular interaction between the components of a system is the appearance in it of a property that was not presented in individual components. In the case of the PEG/TFE telomers system, a switch in surface properties under the influence of thermal heating was detected.

XPS data confirm the transformations occurring in the polymer system and caused by the formation of new bonds and their influence on the properties of the material. Thus, an increase on the surface of the proportion of elements, such as fluorine and carbon associated with fluorine, was detected for Sample 2 compared to Sample 1. This is due precisely to the activation process, during which, due to temperature heating, polyethylene glycol softens and the TFE telomer chains come out.

**Figure 6:**
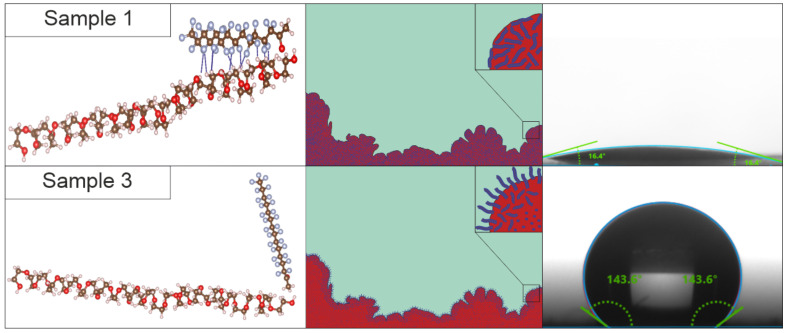
Proposed mechanism for the occurrence of hydrophobic properties in a polymer system.

In the system before activation (Sample 1), the telomer chains, due to the presence of a large number of hydrogen bonds, are at a close distance to the PEG chains (Figure 6). The polyester chains themselves are in a compressed state in the form of globules, which is energetically more favorable (Figure 6). Due to this adhesion of telomers to the polyester, water on the surface of the film of the polymer system comes into contact mainly with the hydrophilic groups of PEG.

In the activated state, polyethylene glycol softens when exposed to heat. Polyester globules unfold due to more active movement of atoms in the molecule (Figure 6), and the telomer chains are affected, which leads to the displacement of TFE telomer molecules to the surface. Due to the displacement of chains onto the surface, a structure of cilia or piles is formed [24] with hydrophobic groups (–CF_3_ and –CF_2_–), which imparts new properties to the surface.

When cooled, the mobility of the PEG molecule in the polymer system decreases. At the same time, the pressure of the polyester chains on the TFE telomers decreases. Due to this, they return close to their original position, and the surface of the film becomes hydrophilic. According to XPS data, on the surface of Sample 2 there are fewer bonded fluorine atoms of the CF_2_ and CF_3_ groups of TFE telomers with hydrogen bonding, which means that the telomer chains more easily move from the “adjacent” position to the formation of a hydrophobic polymer brush.

Subsequently, Sample 2 exhibits the property of switching wettability under the influence of temperature. Moreover, according to DTA data and the wettability of the polymer system, the first signs of a short-term transition begin around 41 °C. The transition of the surface to a hydrophobic state occurs after complete softening of polyethylene glycol closer to 60 °C (Figure 1).

The changes occurring in a polymer system can be described by supramolecular interactions between substances. This is confirmed by the presence of non-covalent bonds between the fluorine atoms of the TFE telomers and PEG. Additionally, the supramolecular system has properties which were not previously presented in individual substances before their interaction. In this case, it is the ability of the polymer system to change the nature of wettability with water under the influence of temperature.

Based on these data, a mechanism was proposed for the interaction that occurs on the surface of the polymer system and leads, when heated, to a change in the properties of the surface. Figure 6 shows the process of switching wettability as a result of the interaction between the molecules of TFE telomers and polyethylene glycol. In this case, a polymer brush formed under the influence of temperature (a similar structure is presented in [25]), consisting of telomer chains raised above the surface, is the reason for the appearance of hydrophobic properties.

**Figure 7:**
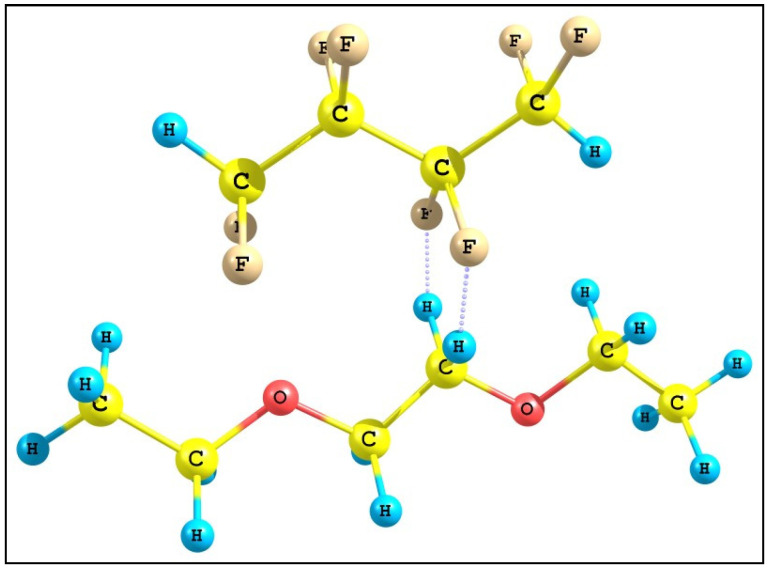
Model of the interaction of octofluoro-butane and diethyl glycol ether used to calculate the energy of the non-covalent fluorine–hydrogen bond.

In order to confirm the proposed hypothesis and on the basis of the data already obtained, a mathematical model of the interaction between the molecules of TFE telomers and PEG was built using compounds similar in structure to the base substances: octofluoro-butane and diethyl glycol ether (Figure 7). The fluorine and hydrogen atoms of these substances form non-covalent bonds. From [24], it is known that the length of the F–H bond is 0.92 Å, and the length of the non-covalent hydrogen bond F⋯H is about 1.4 Å.

For calculations in the model system, the method of functional density theory, functional B3LYP5, basis 6-311 was used. Geometry was optimized separately for each of the elements. Next, the structures were cross-linked at different lengths of the F⋯H hydrogen bond (all carbon atoms are in the same plane). Calculations were carried out for a symmetrical arrangement of atoms according to the C_s_ point group. Hydrogen bond lengths were chosen in the range of 1.40–2.20 Å in steps of 0.2 Å, which covers the range of values presented in studies by different authors. The energy splitting values of the F_1s_ levels are presented in Table 3. To assess the influence of the hydrogen bond on the F_1s_ level, the central CF_2_ and CH_2_ units of each of the compounds used in the model were used. This is necessary to simulate the environment in the polymer corresponding to the samples studied in the experiment [26].

From the data obtained (Table 3), it follows that even the shortest and most energetic hydrogen bond with fluorine is much inferior to the weak hydrogen bond with oxygen. This leads to the fact that, when water comes into contact with the surface of the resulting polymer system, water molecules begin to form hydrogen bonds with PEG molecules, as they are the most energetically favorable. This may be the reason for the destruction of the polymer system in the non-activated state upon contact with water. In the activated state, telomer chains can significantly slow down the rate of this process by limiting the contact area.

The accumulated experience of using polyethylene glycol and its complexes with some substances of polymeric and non-polymeric nature in medicine, as well as a significant number of studies, prove their safety and effectiveness [7,14,15]. This is due to the fact that PEG is a well-known bioresorbable polymer with low immunogenicity, antigenicity and high mobility. Along with polymers such as PLA [27], PLGA [28], polyvinylpyrrolidone [6] PEG actively used as templates for tissue-engineered, protein-activated and gene-activated constructs [29,30,31]. However, the presented system contains chains of tetrafluoroethylene telomers. They are short chains of fluorinated hydrocarbons with active end groups. The polymer itself has strong hydrophobic properties, and its end groups are capable of being separated from the chain, which leads to their polymerization [20,21]. In this regard, to establish the fact of using this polymer system for medical purposes, preliminary preclinical tests are required. Nevertheless, the presented studies allow us to expand our understanding of the possibilities of creating polymer supramolecular systems based on polyethylene glycol, capable of exhibiting smart properties. In this case, TFE telomeres can be replaced with other halogen-substituted hydrocarbons, which can have many more potential biological applications and at the same time retain the possibility of supramolecular interaction with polyester and exhibit improved properties.

## 4. Materials and Methods

### 4.1. Preparation of Polymer System

For the formation of the polymer system, 0.1 g of polyethylene glycol (PEG-1500, Component-reaktiv, Moscow, Russia, CAS 25322-68-3) was added to 5 mL of acetone. Then, with stirring, 3 mL of a 12.5% solution of tetrafluoroethylene telomers in acetone (TFE, “Cherflon”, Federal Research Center of Problems of Chemical Physics and Medicinal Chemistry RAS, Moscow, Russia) was added dropwise to obtain a homogeneous mixture [17].

### 4.2. Preparation of Films

The resulting mass was applied to the surface of a glass slide by spreading. The obtained sample (Sample 1) was left to dry at room temperature for 10 min. Activation of the polymer system was carried out by holding the sample at 70 °C for 5 h and subsequent cooling to room temperature (Sample 2).

The activated system had hydrophilic properties at room temperature. To switch the properties of the system from hydrophilic to hydrophobic, the sample was heated to 60 °C, and then all studies with it were carried out at this temperature (Sample 3).

For comparative analysis, TFE telomer film and PEG film were also prepared. Films were deposited from solutions of these substances in acetone; the concentrations corresponded to those specified in Section 4.1.

### 4.3. Thermal Analysis

Thermal stability of the resulting polymer system was studied by differential thermal analysis (DTA). The studies were carried out on the differential thermal analyzer DTG-60H (Shimadzu, Kyoto, Japan) with a heating rate of 2.5 deg min^−1^ in an air atmosphere.

### 4.4. Wettability Measurements

The wettability of the obtained films was estimated by measuring the contact angle of the sessile droplet deposited on the sample surface using a DSA100 device (Kruss GmbH, Hamburg, Germany). The drop volume was 7 μL. The value of the CA was registered after the drop stabilized on the surface for 60 s.

The studies were carried out on Samples 1 and 3. Deionized water, 0.01 M HCl (pH 2) and 0.01 M NaOH (pH 12) were used as the test liquids.

The surface free energy was calculated by the Owens–Wendt–Rabel–Kaelble (OWRK) method [19]. To determine the CA, deionized water and methylene iodide (CH_2_I_2_) were used, the surface tension of which was established with high accuracy [32]. The dispersed and polar components of the free surface energy of the material were determined by solving the system of two Owens equations for various test liquids:

(1) $$1+\cos\left(\theta \right)=2\sqrt{\gamma_{s}^{d}}\cdot \left(\frac{\sqrt{\gamma_{l}^{d}}}{\gamma_{l}}\right)+2\sqrt{\gamma_{s}^{p}}\cdot \left(\frac{\sqrt{\gamma_{l}^{p}}}{\gamma_{l}}\right),$$

where *θ* is CA between the wetting fluid and the surface; $\gamma_{s}^{d}$ is dispersed component of SFE; $\gamma_{s}^{p}$ is polar component of SFE; $\gamma_{l}$ is the surface energy of the wetting fluid; $\gamma_{l}^{d}$ is the dispersed component of the surface energy of a liquid, and $\gamma_{l}^{p}$ is the polar component of the surface energy of a liquid.

The SFE of the samples under study was calculated by summing the dispersed and polar components using the Fox equation:

(2) $$\gamma_{s}=\gamma_{s}^{d}+\gamma_{s}^{p}$$

### 4.5. Morphological Study

The study of the surface morphology of the samples was carried out using a Sigma 300 VP scanning electron microscope (SEM, Carl Zeiss Group, Jena, Germany) on the basis of the Far Eastern Center for Electron Microscopy of the National Scientific Centre of Marine Biology FEB RAS (Vladivostok, Russia).

The surface topography was studied using an SPM-9600 (Shimadzu Corporation, Kyoto, Japan) scanning probe microscope–atomic force microscope (AFM). The survey was carried out on an area of 6 × 6 µm, with a resolution of 512 × 512 points.

### 4.6. IR Spectroscopy

Attenuated total internal reflection (ATR) IR spectra were recorded in the range 350–4000 cm^−1^ using a Vertex 70v (Bruker, Berlin, Germany) vacuum FTIR spectrometer equipped with a BRUKER Platinum A225 ATR-Einheit ATR attachment with a diamond optical element. ATR spectra were converted into absorption spectra using mathematical processing in standard programs included in the OPUS instrument software.

### 4.7. X-ray Photoelectron Spectroscopy

X-ray photoelectron spectroscopy (XPS) was used for the analysis of the chemical composition of the investigated polymer systems using the SPECS device (SPECS, Berlin, Germany) with the 150 mm hemispherical electrostatic analyzer. Ionization was carried out with non-monochromatized Al Ka (1486.6 eV) radiation. The transmission energy of the analyzer was 50 eV, and the step was 0.1 eV for high resolution spectra and 1 eV for survey spectra. The scale was calibrated using the peaks of C_1s_ hydrocarbons (Eb = 285.0 eV).

## 5. Conclusions

A polymer system based on PEG and TFE telomers was obtained. It has been established that, after temperature activation, the polymer system changes its wettability depending on temperature. At a temperature of 60 °C, the contact angle of the film of the polymer system reaches 143°, decreasing as it cools.

The structural features of the surface layers of the polymer system, and its morphology before and after activation, were established, and a structural model of the surface was proposed that ensures low wettability with water. The supramolecular nature of the polymer system due to hydrogen bonds has been proven.

A mechanism for switching the properties of the polymer is identified and presented, based on a change in the ratio of fluorocarbon and hydroxyl groups on the surface of the material and the formation of a hydrophobic brush-type structure under the influence of temperature.

The synthesized polymer compound, as well as films based on it, can find wide use in medicine, the creation of sensors and other high-tech industries.

### Limitations of the Study

At the moment, the main disadvantage of the polymer system is its insufficient stability when interacting with water. When water reaches the surface of a non-activated polymer system, polyethylene glycol immediately begins to interact with it. Polyester dissolves in an aqueous environment, leaving only hydrophobic telomere chains on the substrate. This is due to the fact that the energy of the supramolecular interaction between TFE telomers and PEG, according to the data presented in the article, is lower than the hydrogen bond with oxygen.

One of the goals of further research is to create a system with stronger connections between components while maintaining its mobility. At the same time, the conditions for the formation of a copolymer based on the system can be much more complex, as well as the possible behavior of the formed system, which will require a whole range of further research activities. At the same time, the data themselves can also be a source of new ideas for the formation of systems with similar properties.

## Figures and Tables

**Table 1:** Values of the surface free energy (*γ*_s_), its dispersive and polar components, as well as the contact angle for the studied polymer films.

Sample	*γ_s_* (mJ/m^2^)	*γ_s_^d^* (mJ/m^2^)	*γ_s_^p^* (mJ/m^2^)
TFE telomers film	6.4 ± 0.7	5.4 ± 0.4	1.0 ± 0.3
PEG film	79.7 ± 0.6	49.7 ± 0.4	30.0 ± 0.2
Sample 1	44.0 ± 3.0	25.9 ± 1.3	17.6 ± 1.4
Sample 2	53.0 ± 6.0	17.3 ± 1.0	36.0 ± 5.0
Sample 3	13.0 ± 2.0	12.0 ± 2.0	1.0 ± 0.1

**Table 2:** Relative content (at. %) and binding energy (eV) of elements in the studied samples.

Sample		Possible Chemical State	Binding Energy (eV)	Content (at.%)
Non-activated	C_1s_	CF_3_	294.1	0.8
		CF_2_	292.4	13.4
		CF_2_⋯H	290.9	7.3
		COC(F) C=O	289.6	2.2
		CO	287.3	2.2
		CH	285.0	6.8
	F_1s_	CF_3_	690.8	3.2
		CF_2_⋯H	689.3	29.0
		CF_2_	687.8	27.5
		FCH	686.3	2.8
	O_1s_	H_2_O	534.5	0.4
		COC(F)	532.9	0.9
		COC	531.1	3.5
Activated	C_1s_	CF_2_	291.5	22.6
		COC(F) C=O	290.2	2.6
		CO	286.9	1.8
		CH	285.0	1.8
	F_1s_	CF_3_	690.2	4.3
		CF_2_⋯H	688.8	29.8
		CF_2_	687.7	30.5
		FCH	686.3	4.0
	O_1s_	H_2_O	534.8	0.1
		COC(F)	533.0	1.0
		COC	531.5	1.5

**Table 3:** Splitting of ∆F_1s_ levels for hydrogen bonds of different lengths.

Bond Length F^…^H (Å)	∆F_1s_ (eV)
2.20	0.13
2.00	0.15
1.80	0.20
1.60	0.28
1.40	0.33

## Data Availability

The data presented in this study are available on request from the corresponding author.
